# Driving Effects of Soil Microbial Diversity on Soil Multifunctionality in *Carya illinoinensis* Agroforestry Systems

**DOI:** 10.3390/microorganisms13112425

**Published:** 2025-10-23

**Authors:** Cheng Huang, Mengyu Zhou, Fasih Ullah Haider, Lin Wu, Jia Xiong, Songling Fu, Zhaocheng Wang, Fan Yang, Xu Li

**Affiliations:** 1 Hubei Key Laboratory of Biological Resources Protection and Utilization, Hubei Minzu University, Enshi 445000, China; 2024102@hbmzu.edu.cn (C.H.);; 2 School of Forestry and Landscape Architecture, Anhui Agricultural University, Hefei 230036, China; 3 National Ecological Science Data Center Guangdong Branch, South China Botanical Garden, Chinese Academy of Sciences, Guangzhou 510650, China; haider281@scbg.ac.cn; 4 School of Cultural Creativity, Anhui Finance & Trade Vocational College, Hefei 230601, China

**Keywords:** agroforestry, soil microorganisms, soil multifunctionality, sustainable management, pecan

## Abstract

Sustainable soil management requires striking a balance between productivity and soil health. While agroforestry practices are known to improve soil health and ecosystem functions, the contribution of microbial diversity to maintaining multifunctional soil processes in pecan (*Carya illinoinensis*) cultivation has yet to be fully elucidated. This study examined microbial diversity, soil functions, and multifunctionality across different pecan intercropping setups. We compared a monoculture pecan plantation with three agroforestry models: pecan–*Paeonia suffruticosa*–*Hemerocallis citrina* (CPH), pecan–*P. suffruticosa* (CPS), and pecan–*P. lactiflora* (CPL). We employed high-throughput sequencing (16S and ITS) to determine the soil bacterial and fungal communities and analyzed the species diversity, extracellular enzyme activities, and physicochemical properties. Soil multifunctionality (SMF) was evaluated using 20 indicators for nutrient supply, storage, cycling, and environmental regulation. Agroforestry increased soil fungal diversity and improved multifunctionality when compared to monoculture. The CPS and CPH models were the most beneficial, increasing multifunctionality by 0.74 and 0.55 units, respectively. Structural equation modeling revealed two key pathways: bacterial diversity significantly enhanced nutrient cycling and environmental regulation, whereas fungal diversity primarily promoted nutrient cycling. These pathways together delivered clear gains in multifunctionality. Random forest analysis identified key predictors (total nitrogen, total carbon, available potassium, β-1,4-N-acetylglucosaminidase, and alkaline phosphatase), highlighting the joint importance of nutrients and microbial enzymes. Our results demonstrate that selecting species in pecan agroforestry alters microbial communities and activates key functions that support soil health and long-term resilience. Hence, pecan agroforestry maintains SMF through microbial processes, with CPS showing the strongest effect. These results can inform species selection and encourage broader testing for resilient, biodiversity-based farming practices.

## 1. Introduction

Soil health, the cornerstone of sustainable agricultural production, is of paramount importance. It is crucial for ensuring the soil performs its functions fully. In recent years, the concept of soil multifunctionality (SMF), centered on soil health and quality, has garnered widespread attention [1,2]. SMF reflects the comprehensive capacity of soil to support the provision of multiple ecosystem functions and services, such as nutrient cycling, organic matter decomposition, and environmental regulation [1,2]. In contrast to traditional soil fertility assessments, SMF characterizes soil functional features through highly sensitive biological, chemical, and physical indicators. However, there remains no unified standard for the methods used to quantify multifunctionality or for the number of indicators employed to reflect it [3]. Most studies agree that SMF primarily encompasses nutrient storage, nutrient cycling, nutrient supply, organic matter decomposition, and soil environmental regulation, thereby providing a more comprehensive reflection of the actual soil condition [2,4]. Microorganisms in soils are essential contributors to nutrient cycling, carbon storage, and the breakdown of contaminants within terrestrial ecosystems, playing a central role in controlling SMF [5,6,7,8,9]. Microbial diversity can underpin ecosystem multifunctionality through mechanisms such as altering resource allocation, enhancing nutrient supply, and mitigating stressful environmental conditions [2,9,10,11]. Changes in soil microbial properties (e.g., diversity and nutrient cycling) are widely recognized as key indicators of ecosystem resilience [12,13]. Biodiversity enhances ecosystem functioning through ecological mechanisms such as niche partitioning and resource use complementarity [14]. However, management disturbances disrupt the stable structure of soil microbial communities, intensifying interspecific competition and consequently altering key microbial taxa. For instance, soil degradation from logging creates new niches associated with oxygen-limited habitats [15]. Furthermore, the depletion of C and N nutrients during extensive forest management reduces microbial populations sensitive to substrate availability [16]. Numerous studies propose that soil microbial diversity, microbial biomass, and extracellular enzyme activity reflect the status of decomposition, nutrient cycling, and overall soil conditions [17,18]. In particular, soil microbial species richness is recognized as a pivotal driver shaping SMF. Findings by Van Rijssel et al. [19] indicated that SMF declines as management intensity increases, with soil organic carbon and bacterial biomass identified as key abiotic and biotic determinants of this multifunctionality. Additionally, the respective impacts of bacterial and fungal communities on soil functions vary depending on the environmental context and land use practices. For example, bacterial diversity has been linked to enhanced SMF in subtropical grasslands and tea cultivation systems [20], while fungal diversity has a more pronounced influence on multifunctionality in dry ecosystems such as those on the Loess Plateau [21].

Although traditional monoculture-based intensive forestry or agriculture has significantly increased short-term yields, such intensive management practices are also recognized as major drivers of soil degradation, imbalanced nutrient depletion, biodiversity loss, soil acidification, and greenhouse gas emissions [22,23]. In contrast, agroforestry systems, as an efficient and ecologically sound form of land use, offer a promising solution. They integrate the practical benefits of traditional agriculture and modern forestry techniques, enabling more efficient utilization of land resources and enhanced soil functionality. These systems have been widely implemented in both China and globally, and research indicates that they enhance ecosystem service delivery and biodiversity by an average of 23% worldwide, with most soil functions in agroforestry systems surpassing those in monoculture forests [24,25]. The potential of agroforestry to enhance soil functionality is significant. Multiple investigations have highlighted that agroforestry intercropping systems outperform monoculture by improving a range of agro-ecosystem services, including yield improvement, soil quality advancement, and increased soil carbon storage [26]. For example, integrating rubber trees (*Hevea brasiliensis*) with salmwood (*Cordia alliodora*) has been found to boost soil organic carbon and total nitrogen by approximately 20% and 14%, respectively [27]. Similarly, tea (*Camellia sinensis*) grown alongside Chinese chestnut (*Castanea mollissima*) orchards has been shown to improve soil nutrient availability and stimulate enzyme activities [28]. In an intercropping system involving poplar (*Populus deltoids*) and wheat (*Triticum aestivum*), higher levels of soil organic matter, essential nutrients, and enzyme activities were reported compared to wheat monoculture [29]. The increased diversity of crops results in a broader spectrum of root exudates, which positively shape the diversity and structure of the soil microbial community, in turn supporting improved soil functions [30,31]. For instance, Li et al. [32] mentioned that, in a rubber-based agroforestry system, enhanced litter and root biomass from intercropping provided substrates that fostered microbial diversity and elevated extracellular enzyme activities, thereby promoting organic matter breakdown and nutrient cycling. Research indicates that plant–microbe interactions within agroforestry systems can drive significant shifts in bacterial community organization and functional potential [33]. However, specific systems, such as those combining Bauple nut (*Macadamia integrifolia*) with crops like maize (*Zea mays*) and konjac (*Amorphophallus konjac*), have been reported to reduce bacterial network complexity and increase soil compaction, leading to decreases in soil multifunctionality [6]. Thus, the impact of agroforestry on soil microbial diversity is highly variable and depends mainly on the choice of understory species and management intensity [27]. Furthermore, studies indicate a feedback regulation between changes in soil characteristics and the structure and function of soil microbial communities. For example, increases in total organic carbon (TOC) and total nitrogen (TN), along with a suitable pH range, have been shown to enhance bacterial community diversity significantly [34]. This increased species diversity may promote the decomposition of recalcitrant soil organic matter, thereby improving nutrient availability [26]. It has also been reported that soil moisture, the abundance of soil aggregates, and soil depth significantly influence microbial community assembly and the composition of functional groups [16]. Within agroforestry systems, tillage disturbance and the management of intercrops variably impact soil physical structure and chemical properties, changes which are certain to alter the species composition of the soil microbial community.

The pecan tree (*Carya illinoinensis*), originally native to North America, is now extensively cultivated in over 20 countries, including the United States, Mexico, South Africa, and China [35]. Over the past decade, it has been extensively introduced and cultivated across central and eastern provinces of China [26]. Due to its long juvenile phase and tall tree structure with spacious underground areas, pecan trees have been intercropped with various crops throughout the country in different configurations to improve land use efficiency and generate early-stage income before nut production [36]. Maintaining and enhancing SMF within pecan-based agroforestry systems is recognized as vital for promoting sustainable plantation productivity and ecosystem resilience. While previous studies have demonstrated that agroforestry can positively influence soil microbial diversity and associated ecosystem functions, the specific mechanisms by which soil microbial communities drive SMF in pecan agroforestry systems remain poorly understood. Moreover, the responses of microbial diversity and soil functions to different intercropping configurations, particularly in pecan plantations, have not been comprehensively evaluated. This represents a critical knowledge gap, given the increasing adoption of pecan intercropping to optimize land use and in-crease economic returns before nut production.

To address this gap, the current study compared soil microbial diversity and multifunctionality across three representative pecan-based agroforestry practices, using a monoculture pecan plantation as a control. The main objectives were: (1) to evaluate how different intercropping systems affect soil bacterial and fungal diversity; (2) to determine the influence of these microbial changes on multiple soil functional dimensions; and (3) to identify management strategies and species combinations that optimize SMF in pecan agroforestry systems. We hypothesized that agroforestry configurations with complementary understory species enhance microbial diversity, thereby improving nutrient cycling, storage, and overall SMF compared to monocultures. This study’s results aim to uncover the underlying microbial mechanisms that drive functional processes in pecan agroforestry systems, guiding sustainable management strategies that support soil health, enhance ecosystem services, and ensure the long-term productivity of pecan orchards.

## 2. Materials and Methods

### 2.1. Study Area

The research was conducted at a pecan orchard owned by Fuyang Xinfeng Seed Co., Ltd., located in Yingquan District, Fuyang City, Anhui Province (coordinates: 115°33′44″ E, 32°57′25″ N). The area experiences a warm, semi-humid monsoon climate, characterized by annual rainfall ranging from 750 to 900 mm, an average temperature of 15 °C, and an elevation of approximately 33 m (China Meteorological Data Service, http://data.cma.cn/). The predominant soils are Vertisols which are classified as uderts (suborder) according to soil taxonomy [37] (thickness ranges from 50 cm to 150 cm, pH ranges from 7.5 to 8.5), characterized by an organic matter (OM) content of 17.5 g kg^−1^, a total nitrogen (TN) content of 1.02 g kg^−1^, available phosphorus (AP) at 17.2 mg kg^−1^, and available potassium (AK) at 178.7 mg kg^−1^.

### 2.2. Experimental Design

Pecan trees of the ‘Pawnee’ cultivar were established there in 2016. Beginning in 2017, three understory species, *Paeonia × suffruticosa*, *Hemerocallis citrina*, and *Paeonia lactiflora*, were planted beneath the pecan canopy. In September 2019, the experiment was conducted using a randomized block design with four treatments: three agroforestry intercropping models and a monoculture pecan plantation, which served as the control (CK), resulting in a total of 12 experimental plots. Each treatment had three replicates, with each plot covering more than 2 hectares. Pecan trees were spaced at 4 m by 6 m. The agroforestry treatments included (1) pecan with *Paeonia × suffruticosa* and *Hemerocallis citrina*—spacing for *Paeonia × suffruticosa* was 0.2 m by 0.2 m, and for *Hemerocallis citrina* was 0.4 m by 0.8 m; (2) pecan with *Paeonia × suffruticosa* (CPS) with spacing 0.2 m by 0.6 m; and (3) pecan with *Paeonia lactiflora* (CPL) spaced at 0.2 m by 0.6 m. Throughout the study, all plots had consistent irrigation and fertilization. Using sensor detection, the drip irrigation system automatically activates when the soil moisture content in the 0–20 cm layer drops below 15% between March and November, and ceases operation when the moisture content reaches 40%. Irrigation is suspended from December to February. Organic fertilizer (45% organic matter content, mainly composed of fermented sheep manure particles) was applied at a rate of 15,000 kg per hectare in winter, after leaf drop, while compound fertilizer was used in the summer.

### 2.3. Sampling Design

In early September 2019, soil samples were collected from the experimental plots. Before sampling, surface vegetation and litter were removed. Using a soil core sampler, samples were taken at a depth of 0–20 cm from five distinct locations within each plot, resulting in a total of 12 composite samples. After collection, the subsamples from each plot were combined and passed through a 2-mm sieve to remove fine roots and gravels, forming a composite sample for microbiological and physicochemical analyses. These composite samples were stored in insulated containers at 4 °C and transported quickly to the laboratory under cooled conditions for further processing.

### 2.4. Laboratory Analysis

#### 2.4.1. Physicochemical Analysis

Soil bulk density (BD), moisture content (MC), and porosity (SP) were measured using the gravimetric technique. Soil pH was determined in a 1:2.5 soil-to-water suspension employing a pH meter (Mettler Toledo, FE28-Standard, Zurich, Switzerland). Concentrations of nitrate nitrogen (NO_3_^−^-N), ammonium nitrogen (NH_4_^+^-N), AP, and AK were quantified with an automated discrete chemical analyzer (CleverChem Anna, DeChem-Tech, Germany). Total carbon (TC) and TN contents were analyzed using an elemental analyzer (Vario EL Cube, Elementar, Langenselbold, Germany), while total potassium (TK) was measured via inductively coupled plasma optical emission spectrometry (iCAP 6300 series, Thermo Fisher Scientific, Waltham, MA, USA).

#### 2.4.2. Enzyme Activity Assays

Fresh soil samples were air-dried and sieved through a 50-mesh screen before further analysis. The activities of soil enzymes, including urease (UE), alkaline phosphatase (AKP), cellobiohydrolase (CBH), 1,4-β-N-acetylglucosaminidase (NAG), β-1,4-glucosidase (BG), polyphenol oxidase (PPO), and peroxidase (POD), were determined using visible spectrophotometry with reagent kits supplied by Shanghai Preferred Bio-Technology Co., Ltd. (Shanghai, China) [26]. The specific kit catalog numbers used were as follows: YX-C-B933 for UE, YX-C-B902 for AKP, YX-C-SC1 for CBH, YX-C-SNAG for NAG, YX-C-B935 for BG, YX-C-B934 for PPO, and YX-C-B914 for POD.

#### 2.4.3. Illumina MiSeq Amplicon Sequencing of 16S rDNA and ITS2 Region

Soil microbial communities were characterized using Illumina MiSeq high-throughput sequencing. Total microbial DNA was extracted from 0.5 g of fresh soil samples with the E.Z.N.A.^®^ Soil DNA Kit (D4015, Omega, Inc., Norcross, GA, USA) following the manufacturer’s instructions. DNA purity was verified by 2% agarose gel electrophoresis, and ultra-pure water was included as a negative control to exclude potential false-positive PCR results. After assessing purity and concentration, the V3–V4 region of bacterial 16S rRNA was amplified via PCR using the primers 341F (5′-CCTACGGGNGGCWGCAG-3′) and 805R (5′-GACTACHVGGGTATCTAATCC-3′). Fungal ITS2 regions were amplified using primers fITS7 (5′-GTGARTCATCGAATCTTTG-3′) and ITS4 (5′-TCCTCCGCTTATTGATATGC-3′) [16]. The 5′ ends of all primers were tagged with sample-specific barcodes and universal sequencing adapters.

The PCR conditions for prokaryotic 16S amplification were as follows: initial denaturation at 98 °C for 30 s; 25 cycles of denaturation at 98 °C for 12 s, annealing at 54 °C for 30 s, and extension at 72 °C for 45 s, followed by a final extension at 72 °C for 10 min. Amplicons were confirmed by 2% agarose gel electrophoresis. Throughout DNA extraction and PCR, ultra-pure water was used as a negative control to monitor contamination. PCR products were purified using AMPure XT beads (Beckman Coulter Genomics, Danvers, MA, USA) and quantified with a Qubit fluorometer (Invitrogen, Carlsbad, CA, USA). Amplified libraries were assessed for size and quantity using an Agilent 2100 Bioanalyzer (Agilent, Santa Clara, CA, USA) and the Illumina Library Quantification Kit (Kapa Biosciences, Wilmington, NC, USA). A total of 258,748 and 428,470 high-quality sequences were obtained for bacteria and fungi, respectively, with median read counts per sample of 17,889 (range: 14,374–61,886) for bacteria and 20,427 (range: 14,672–87,915) for fungi.

Libraries were sequenced on the NovaSeq PE 250 platform. Chimeric sequences were filtered using Vsearch (v2.3.4), and amplicon sequence variants (ASVs) were derived using DADA2 [38]. Taxonomic classification of bacterial and fungal ASVs was performed against the SILVA (https://www.arb-silva.de/) (accessed on 25 October 2019) and UNITE (https://unite.ut.ee/) (accessed on 27 October 2019) databases, respectively. Alpha diversity (Chao1, Shannon–Wiener, and Simpson indices) was calculated to evaluate within-sample diversity. Beta diversity was assessed using non-metric multidimensional scaling (NMDS) based on Bray–Curtis distances, and statistical significance among groups was tested with permutational multivariate analysis of variance (PerMANOVA). Identification of differentially abundant taxa (genus level) was performed using the Kruskal.test. All bioinformatic analyses were conducted using the OmicStudio platform (https://www.omicstudio.cn/tool) (accessed on 29 October 2019).

### 2.5. Data Analysis

Based on previously established methodologies [3,39], four key soil functions were selected to reflect soil multifunctionality: nutrient supply, nutrient storage, nutrient cycling, and environmental regulation (Table 1).

The ecosystem multifunctionality index was computed using both the single-function approach and the averaging method. All measured functional indicators were standardized using Z-score normalization:

(1) $$Z_{\mathrm{ij}}=\;\;\frac{\left(X_{ij}\;-\;\mu_{j}\right)}{\delta_{j}}$$

where *Z_ij_* represents the Z-score of the *j*-th soil function indicator in the *i*-th plot, *X_ij_* denotes the measured value of the *j*-th soil function indicator in the *i*-th plot, *μ_j_* is the mean value of the *j*-th soil function indicator across all plots, and *δ_j_* is the standard deviation of the *j*-th soil function indicator across all plots.

The single-function index was calculated as follows:

(2) $$F_{\mathrm{ij}}=\;\;\frac{\sum_{j}^{n}Z_{ij}}{n}$$

where *F_ij_* represents the functional index of the *j*-th function in the *i*-th plot, and *n* denotes the number of functional indicators included in that function.

The soil multifunctionality index was calculated using the following formula:

(3) $$\mathrm{SMF}\;=\;\;\frac{\sum_{j}^{15}Z_{ij}}{20}$$

where SMF denotes the multifunctionality index of the *i*-th plot, and 20 represents the total number of soil functional indicators included in the soil multifunctionality evaluation in this study.

Data were organized using Excel 2019. Mean values and standard deviations of all functional indicators were calculated with SPSS 26.0. One-way analysis of variance (One-Way ANOVA) was employed to examine the effects of agroforestry management patterns on soil microbial diversity and soil functions, followed by Least Significant Difference (LSD) post hoc tests to assess differences in soil functions among different stands. Regression analysis was performed between individual functional indicators and soil multifunctionality in R 4.1.3 using the “basicTrendline” package. Pearson correlation analysis and the Mantel test were employed to quantify the correlations between soil nutrients, enzyme activities, and soil microbial diversity. Graphs were plotted in R 4.1.3 using the “ggplot2”, “vegan”, “dplyr” and “ggcor” packages. Key factors influencing soil multifunctionality were identified based on the criterion of an increase in mean squared error (MSE) < 0.01 using random forest analysis. A structural equation model (SEM) was constructed in AMOS 24.0, incorporating the selected key factors to test the hypothesized pathways through which soil microbial diversity affects soil multifunctionality.

## 3. Results

### 3.1. Changes in Soil Microbial Diversity

**Figure 1:**
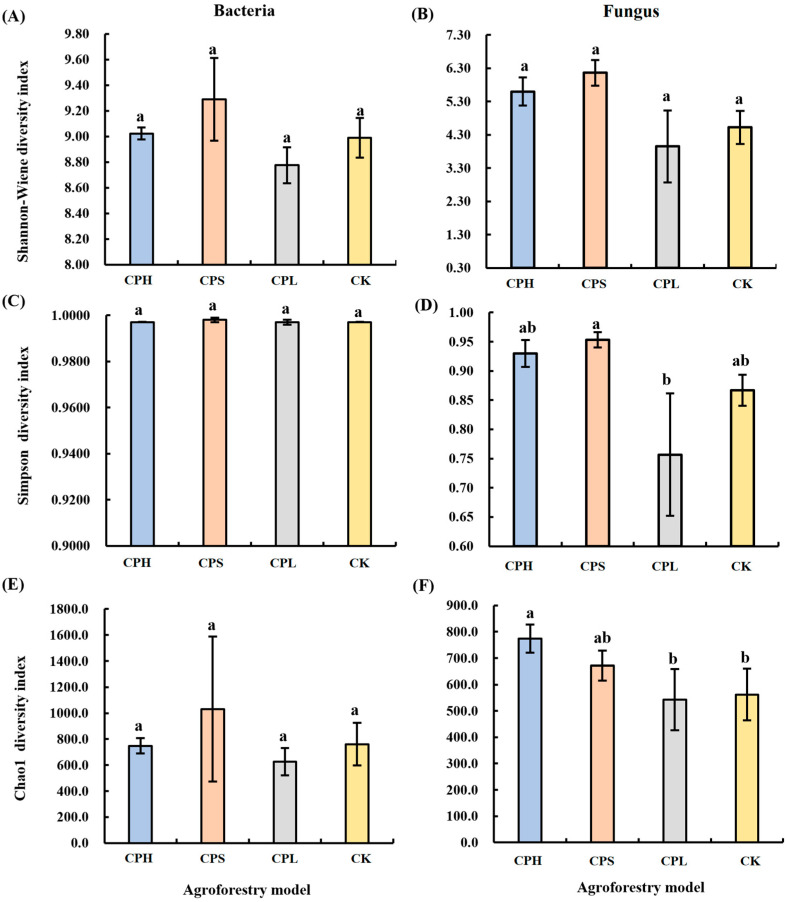
Changes in soil bacterial (**A**,**C**,**E**) and fungal (**B**,**D**,**F**) community diversity under different agroforestry systems. CK = monoculture pecan; CPS = pecan–*Paeonia suffruticosa*; CPH = pecan–*P. suffruticosa*–*Hemerocallis citrina*; CPL = pecan–*P. lactiflora*. Different lowercase letters indicate significant differences among treatments at *p* < 0.05. Significance levels: *p* < 0.05, *p* < 0.01, *p* < 0.001.

No significant differences were observed in soil bacterial diversity across the different agroforestry practices. However, in contrast to bacterial diversity, significant differences were detected in fungal diversity among the management regimes (Figure 1). Specifically, the CPS system showed the highest Simpson diversity index for fungal communities, which was 9.44% higher than that in the CK, followed by the CPH system. By comparison, the CPL system resulted in a significantly lower fungal Simpson diversity index compared to CK. Furthermore, regarding fungal richness, the Chao1 index was highest under the CPH system, being 36.93% higher than that in CK and significantly exceeding both the CK and CPL systems.

**Figure 2:**
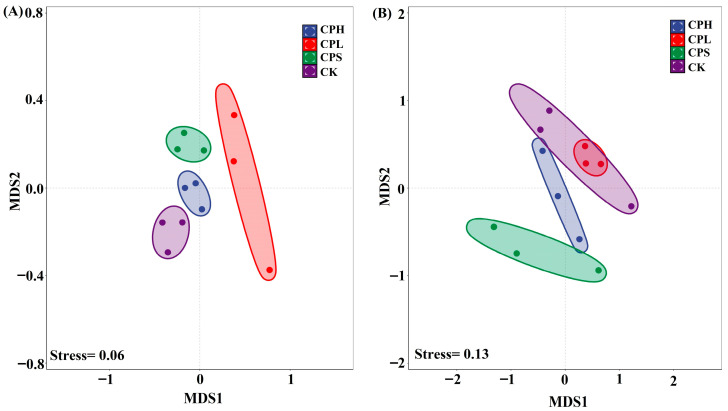
Non-metric Multidimensional Scaling (NMDS) of soil bacterial (**A**) and fungal (**B**) community composition.

The non-metric multidimensional scaling (NMDS) results indicate a good model fit for both soil bacterial (Stress = 0.06) and fungal (Stress = 0.13) community distributions (Figure 2). Consistent with the α-diversity analysis, PerMANOVA results demonstrated no significant differences in soil microbial composition across the different agroforestry management patterns when compared to the control (Table S1).

**Figure 3:**
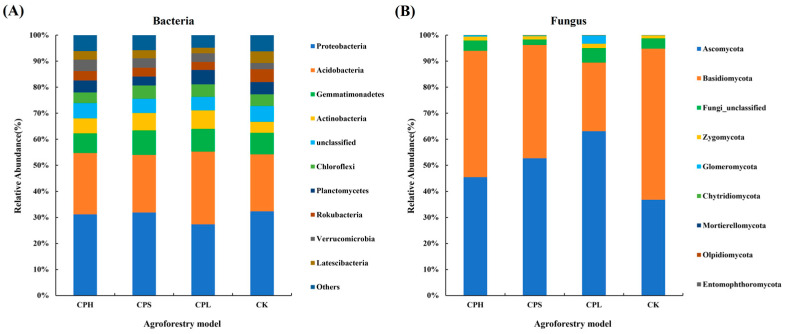
Characteristics of relative abundance of dominant soil bacterial (**A**) and fungal (**B**) communities (phylum level) under different agroforestry patterns.

The dominant bacterial phyla with relatively high abundance in soil included *Proteobacteria* (27.35–32.32%), *Acidobacteria* (21.87–23.51%), *Gemmatimonadetes* (7.54–9.42%), *Actinobacteria* (4.26–7.14%), and *Chloroflexi* (4.10–5.10%). The dominant fungal phyla were *Ascomycota* (45.43–63.08%), *Basidiomycota* (26.40–58.05%), and *Zygomycota* (1.06–1.72%) (Figure 3). Compared to CK, the composition of dominant bacterial phyla remained relatively consistent across different agroforestry systems. However, it is noteworthy that the relative abundance of *Glomeromycota* was higher under the CPL treatment, while *Basidiomycota* was less abundant under the CK treatment. The Kruskal–Wallis test results indicated that the bacterial phyla *Acidobacteria*, *GN04*, *Verrucomicrobia*, *Omnitrophicaeota*, *Actinobacteria*, and *WS3* showed significant differences (*p* < 0.05) among the agroforestry systems. In contrast, among fungi, only *Glomeromycota* exhibited a significant difference (*p* < 0.05) at the phylum level (Tables S3 and S4).

Correlation analysis indicated that, among the dominant bacterial phyla, *Proteobacteria* was significantly negatively correlated with EC and NO_3_^−^-N (*p* < 0.05), while *Acidobacteria* showed significant positive correlations with EC and NO_3_^−^-N (*p* < 0.05). *Gemmatimonadetes* was positively correlated with UE (*p* < 0.05) but negatively correlated with PPO (*p* < 0.05). *Actinobacteria* exhibited significant positive correlations with EC, UE, and AKP (*p* < 0.05) and a negative correlation with POD (*p* < 0.05). *Chloroflexi* was negatively correlated with PPO (*p* < 0.05), whereas *Planctomycetes* was positively correlated with NO_3_^−^-N (*p* < 0.05) and negatively correlated with NAG (*p* < 0.05). *Rokubacteria* was negatively correlated with TN, UE, and AKP (*p* < 0.05) but positively correlated with POD (*p* < 0.05). *Verrucomicrobia* showed significant positive correlations with AP, AK, TN, TC, AKP, and NAG (*p* < 0.05) and a negative correlation with CBH (*p* < 0.05). Additionally, *Latescibacteria* was extremely significantly negatively correlated with UE (*p* < 0.01) and positively correlated with POD (*p* < 0.01) (Figure S1). In contrast, fungal communities demonstrated weaker correlations with soil physicochemical properties and enzyme activities. Specifically, *Ascomycota* was only positively correlated with TP (*p* < 0.05), *Basidiomycota* was negatively correlated with TP (*p* < 0.05), *Glomeromycota* was positively correlated with EC (*p* < 0.05), *Chytridiomycota* was positively correlated with UE (*p* < 0.05), *Mortierellomycota* was negatively correlated with POD (*p* < 0.05), and *Entomophthoromycota* was negatively correlated with EC, TN, and AKP (*p* < 0.05) but positively correlated with CBH (*p* < 0.05) (Figure S2).

### 3.2. Changes in Soil Functional Characteristics

**Figure 4:**
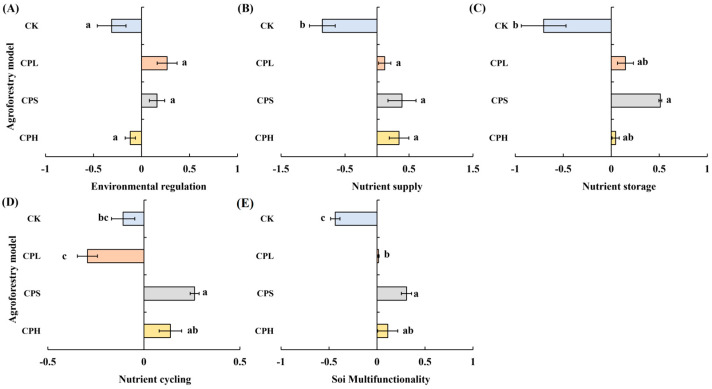
Soil multifunctionality under different agroforestry systems. CK = monoculture pecan; CPS = pecan–*Paeonia suffruticosa*; CPH = pecan–*P. suffruticosa–Hemerocallis citrina*; CPL = pecan–*P. lactiflora*. Different lowercase letters indicate significant differences among treatments at *p* < 0.05.

**Figure 5:**
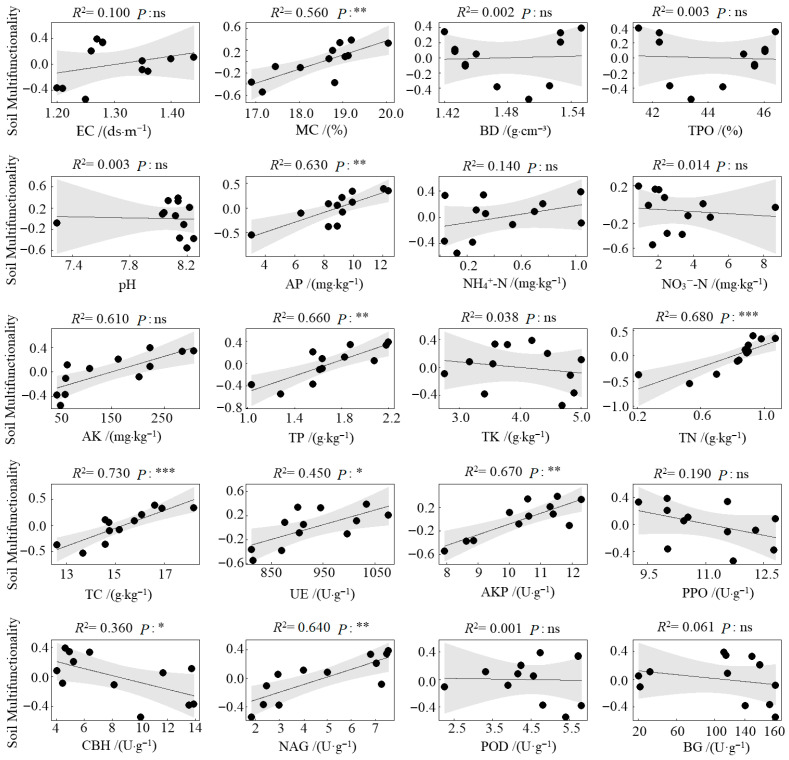
Linear relationship between soil functional indicators and soil multifunctionality. ***, *p* < 0.001; **, *p* < 0.01; *, *p* < 0.05; ns, not significant.

Compared to the CK (monoculture pecan plantation), no significant differences were observed in soil environmental regulation function under the different agroforestry systems. However, significant enhancements were detected in nutrient supply, nutrient storage, and nutrient cycling functions (Figure 4). Specifically, all three agroforestry practices significantly strengthened the soil nutrient supply function relative to CK (*p* < 0.05). Soil nutrient storage was also significantly improved, with the CPS system showing markedly higher values than CK (*p* < 0.05). The CPS system similarly exhibited the highest nutrient cycling function, significantly surpassing CK. In contrast, the CPL system resulted in significantly lower nutrient cycling compared to both CPS and CPH systems (*p* < 0.05) and was also inferior to CK. Regarding overall soil multifunctionality, all agroforestry systems significantly outperformed the monoculture control. The CPS system achieved the highest multifunctionality index, exceeding CK by 0.74, followed by CPH, which exceeded CK by 0.55. Results from the linear regression analysis between soil functional indicators and soil multifunctionality revealed that, among the 20 indicators examined, MC, AP, AK, TP, TN, TC, AKP, and NAG showed a highly significant positive correlation with soil multifunctionality (*p* < 0.01). UE was significantly positively correlated with multifunctionality (*p* < 0.05), whereas CBH exhibited a significant negative correlation (*p* < 0.05) (Figure 5).

### 3.3. Effects of Soil Microorganisms on Soil Multifunctionality

**Figure 6:**
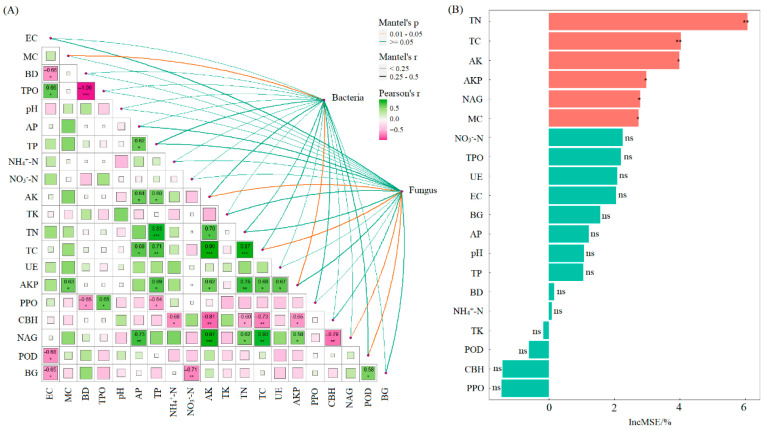
Mantel test between soil microbial diversity and soil indicators (**A**); random forest analysis of effects of soil functional indicators on soil multifunctionality (**B**). Note: MC: soil moisture content; BD: bulk density; TPO: total porosity; EC: electrical conductivity; AP: available phosphorus; AK: available potassium; NH4^+^-N: ammonium nitrogen; NO_3_^−^-N: nitrate nitrogen; TC: total carbon; TN: total nitrogen; TP: total phosphorus; TK: total potassium; UE: urease; AKP: alkaline phosphatase; CBH: cellobiohydrolase; NAG: β-1,4-N-acetylglucosaminidase; BG: β-1,4-glucosidase; PPO: polyphenol oxidase; POD: peroxidase. ***, *p* < 0.001, **, *p* < 0.01; *, *p* < 0.05; ns, not significant.

Pearson correlation analysis and Mantel test results indicated that soil bacterial diversity was significantly correlated with SMC and AKP activity (*p* < 0.05). Soil fungal diversity showed significant correlations with TC, AK, NAG activity, and POD activity (*p* < 0.05) (Figure 6). Random forest analysis further identified that, among the 20 soil functional indicators evaluated, TN and TC had extremely significant effects on soil multifunctionality (*p* < 0.01). Additionally, AK, AKP, NAG, and SMC also significantly influenced multifunctionality (*p* < 0.05).

**Figure 7:**
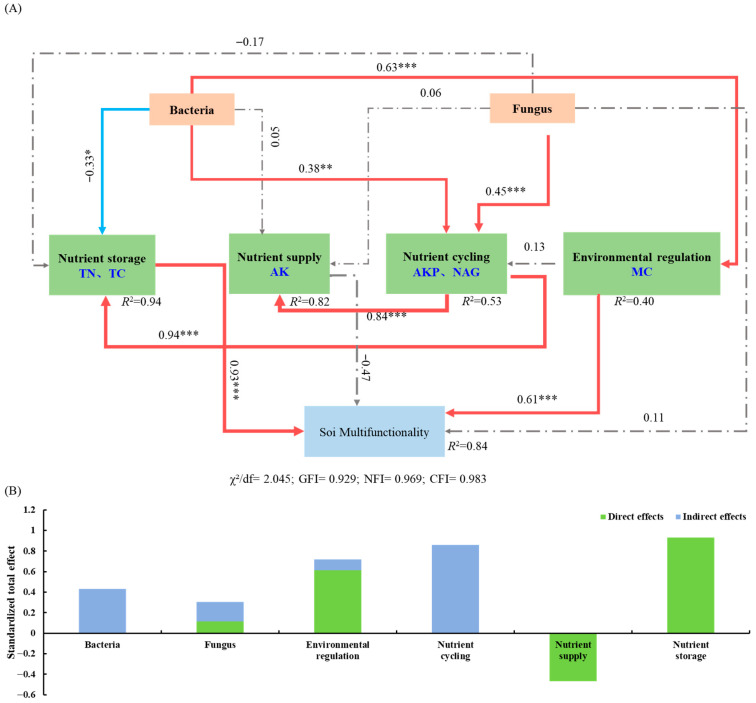
The regulatory pathways of soil microbial diversity on soil multifunctionality and its direct and indirect. Structural equation modeling (**A**), direct and indirect effects of variables (**B**). Note: The red solid line indicates a significant positive effect, the blue solid line indicates a significant negative effect, and the gray dashed line indicates no significant effect. ***, *p* < 0.001; **, *p* < 0.01; *, *p* < 0.05.

The structural equation model (SEM) explained 84% of the variation in soil multifunctionality in the pecan agroforestry systems (Figure 7A). Soil bacterial diversity showed a significant positive effect on nutrient cycling (r = 0.63, *p* < 0.001) and environmental regulation (r = 0.38, *p* < 0.01), but a significant negative effect on nutrient storage (r = −0.33, *p* < 0.05). Soil fungal diversity exhibited a significant positive relationship with nutrient cycling (r = 0.45, *p* < 0.05). Nutrient cycling had significant positive effects on both nutrient supply (r = 0.84, *p* < 0.01) and nutrient storage (r = 0.94, *p* < 0.01). Soil microbial diversity influenced soil multifunctionality indirectly through its effects on nutrient storage, nutrient cycling, and environmental regulation, with bacterial diversity having a stronger overall influence than fungal diversity (Figure 7B). The total effects analysis revealed that nutrient storage (r = 0.93, *p* < 0.001) and environmental regulation (r = 0.61, *p* < 0.001) had significant direct positive effects on soil multifunctionality, while nutrient cycling function primarily exerted indirect effects.

## 4. Discussion

### 4.1. Agroforestry Practices Enhanced Soil Microbial Diversity

The different agroforestry practices altered soil microbial diversity. Conventional theory posits that agroforestry systems enhance plant diversity and thereby positively influence soil microbial diversity; however, our study presents a divergent finding. Specifically, agroforestry did not significantly affect soil bacterial diversity (Figure 1), nor did it induce significant differences in species composition, a result that aligns with recent similar studies [40,41]. One potential explanation for this outcome is the differences in ecological niches among microbial taxa. Compared to fungal communities, bacterial communities occupy a broader range of ecological niches and display greater adaptability to environmental changes. As a result, the shifts in resource availability caused by agroforestry management did not significantly alter resource competition within bacterial communities, thereby maintaining relatively stable levels of bacterial diversity [42]. The relatively short establishment period of the agroforestry system in this study may not have allowed for the full development of distinct microbial communities, which could be another key reason for the lack of significant soil microbial diversity characteristics. This possibility merits continued monitoring. Additionally, it is worth noting that some studies have suggested that soil bacterial diversity does not correlate with plant species richness [43]. Our findings support this notion, as the CPH system exhibited greater plant community richness compared to CK and the other two agroforestry systems; however, it did not demonstrate a significant advantage in microbial diversity. For example, both bacterial and fungal Shannon diversity indices were lower in CPH than in the CPS system. Furthermore, another study reported that, when soybean was used as the main intercropped species, soil microbial diversity in the agroforestry system significantly increased [44]. These results collectively suggest that the critical factor influencing soil microbial diversity may be the selection of specific intercropped plants, rather than merely the number of species present. Moreover, it has been proposed that secondary metabolites, such as organic acids, sugars, and phenolics, exuded by the roots of understory plants, can negatively affect the composition, abundance, and growth of soil microbial communities [45]. In our study, both bacterial and fungal diversity were reduced to varying degrees in the CPL system compared to CK. Although no existing studies have directly reported the inhibitory effects of *Paeonia lactiflora* on soil microorganisms, this observed reduction may be linked to the unique root exudates of this species. This observation suggests an area for further experimental validation.

### 4.2. Agroforestry Practices Enhanced Soil Functions

Compared to the CK, agroforestry practices led to a significant enhancement of soil functions. More intensive soil management improved the soil environment and, to some extent, altered the availability of soil nutrients, as evidenced by characteristic changes in nutrient cycling and storage functions (Figure 2). Furthermore, the soil C:N ratio across the three agroforestry patterns (ranging from 16.98 to 17.67) was found to be lower than that in CK (23.80), which further confirms more efficient soil organic matter accumulation under agroforestry management (Table S2). The multi-layered structure of the pecan tree canopy and lower crops in agroforestry systems increased rainwater interception and reduced evaporation, thereby raising soil moisture content [36]. Linear regression analysis revealed that higher soil moisture had a positive influence on soil multifunctionality (Table S2, Figure 3). Studies indicate that intercropped crops with denser root systems can enhance soil stabilization, reduce erosion, and help maintain soil fertility [24]. It has been suggested that fungal diversity is a key driver of soil multifunctionality, and that intensive agricultural practices can weaken the connection between modular characteristics of microbial networks and individual soil functions, thereby reducing overall soil multifunctionality [46]. Our findings corroborate this, as the CPL treatment showed significantly lower soil multifunctionality compared to the control and other agroforestry systems, alongside the lowest fungal diversity. Furthermore, the relative abundance of Basidiomycota, a phylum known to play a critical role in material decomposition and soil fertility enhancement, was notably reduced under the CPL regime [47,48]. Extracellular enzymes from microbes and root exudates act as catalysts for converting organic compounds into inorganic nutrients [49,50]. We speculate that this decline in Basidiomycota may restrict the secretion of related extracellular enzymes, ultimately leading to decreased efficiency in decomposition and nutrient cycling. Previous research demonstrates that increased enzyme activity improves soil quality in pecan plantations [26]. Although the functions of soil differed significantly among agroforestry systems, they contributed positively to overall soil multifunctionality, supporting earlier findings [40,51]. Therefore, this study suggests, in line with existing literature, that agroforestry practices can positively, neutrally, or negatively affect soil functions, depending mainly on management intensity [25].

We emphasize that pecan agroforestry not only enhances land use efficiency in forestry production but, more importantly, better sustains soil multifunctionality, which is crucial for sustainable forestry. In selecting intercrop species, full consideration should be given to the interspecific relationships with pecans, rather than focusing solely on the economic value of the intercrops; it is better to leverage the ecological advantages of the agroforestry system.

### 4.3. Indirect Effects of Soil Microbial Diversity on Soil Multifunctionality

Microbial communities play a crucial role in regulating ecosystem multifunctionality across diverse habitats [52]. Changes in soil microbial richness and network complexity are associated with higher soil multifunctionality [53]. Studies have shown that rational spatiotemporal distribution of crops can improve the microclimate and nutrient accumulation in the rhizosphere, enhance soil enzyme activities, regulate microbial nutrient metabolism balance, and promote nutrient cycling [54,55]. Results from SEM indicated that increased bacterial and fungal diversity stimulated the secretion of enzymes such as AKP and NAG, thereby facilitating the decomposition of organic matter and improving soil nutrient cycling. Contrary to most previous reports, this study found that increased bacterial diversity had a significant negative effect on soil nutrient storage. This may be related to the balance between carbon and nitrogen fixation and decomposition. Higher bacterial diversity may accelerate the mineralization of soil carbon and nitrogen, making these nutrients more available for plant uptake, thereby potentially reducing the TC and TN content in the soil [56]. However, further research is needed to quantify the expression of microbial genes involved in carbon and nitrogen cycling and to measure the products of organic matter mineralization. Compared to soil fungi, bacteria exerted a stronger influence on soil multifunctionality, primarily through direct effects on nutrient storage and environmental regulation, which aligns with the findings of Wang et al. [57]. This study demonstrated that TC, TN, and SMC had significant positive effects on soil multifunctionality. Therefore, during soil management, it is recommended to supplement nutrients appropriately (e.g., through organic amendments) and maintain adequate soil moisture levels. These measures can not only directly enhance soil multifunctionality but also promote microbial colonization, establishing a positive feedback loop.

### 4.4. Limitations and Future Prospectives

While this study provides valuable insights for the development of pecan agroforestry, several limitations should be acknowledged. First, the single-site nature of the research limits the generalizability of the findings to broader practical applications. Therefore, future studies should establish experimental sites across larger geographical scales and incorporate more diverse agroforestry management patterns to enhance the applicability of the results. Second, in terms of research duration, extending the temporal scope would allow for a more comprehensive understanding of the assembly processes of soil microbial communities. Furthermore, while the current study employed high-throughput sequencing to investigate the effects of taxonomic composition and species diversity on soil multifunctionality, it did not explore microbial functional traits, particularly differences in the expression of key functional genes involved in critical metabolic processes such as carbon, nitrogen, and phosphorus cycling. Consequently, subsequent research should employ more precise experimental methods, such as quantitative PCR and metagenomics, to delve deeper into the ecological processes and co-occurrence patterns of soil microbial communities in agroforestry systems.

## 5. Conclusions

Agroforestry practices enhanced soil microbial diversity and contributed to the maintenance of soil multifunctionality. In the pecan-based agroforestry systems, soil functions, including environmental regulation, nutrient cycling, nutrient supply, and nutrient storage, were improved to varying degrees. Increased soil microbial diversity played a positive indirect role in supporting multifunctionality. Among the three intercropping systems evaluated, the CPS model exhibited higher microbial diversity and the greatest soil multifunctionality, demonstrating high potential for sustainable management.

## Figures and Tables

**Table 1:** Indicators used to evaluate soil multifunctionality (SMF) across nutrient supply, storage, cycling, and environmental regulation.

Functions	Functional Factor
Environmental regulation	MC, BD, TPO, EC, pH
Nutrient supply	AP, AK, NH_4_^+^-N, NO_3_^−^-N
Nutrient storage	TC, TN, TP, TK
Nutrient cycling	UE, AKP, CBH, NAG, BG, PPO, POD

Note: MC: soil moisture content; BD: bulk density; TPO: total porosity; EC: electrical conductivity; AP: available phosphorus; AK: available potassium; NH4^+^-N: ammonium nitrogen; NO3^−^-N: nitrate nitrogen; TC: total carbon; TN: total nitrogen; TP: total phosphorus; TK: total potassium; UE: urease; AKP: alkaline phosphatase; CBH: cellobiohydrolase; NAG: β-1,4-N-acetylglucosaminidase; BG: β-1,4-glucosidase; PPO: polyphenol oxidase; POD: peroxidase.

## Data Availability

The original data presented in the study are openly available in NCBI BioProject database under accession number (SUB15715012).

## Supplementary Materials

The following supporting information can be downloaded at: https://www.mdpi.com/article/10.3390/microorganisms13112425/s1, Figure S1: Correlation between dominant bacterial taxa (phylum level) and soil physicochemical properties and enzyme activities; Figure S2: Correlation between dominant fungal taxa (phylum level) and soil physicochemical properties and enzyme activities; Table S1: PERMANOVA test of soil microbial community species composition under different agroforestry models; Table S2: Soil physicochemical characteristics under different agroforestry model; Table S3: Kruskal-Wallis test-based analysis of differential abundance in bacterial phyla; Table S4: Kruskal-Wallis test-based analysis of differential abundance in fungal phyla.
