# Supplementary material for: Driving Effects of Soil Microbial Diversity on Soil Multifunctionality in Carya illinoinensis Agroforestry Systems
Source: Microorganisms. 2025 Oct 23;13(11):2425. doi: 10.3390/microorganisms13112425 (PMC12654798; doi:10.3390/microorganisms13112425)
Supplement: Supplementary file 1 [file microorganisms-13-02425-s001.zip › microorganisms-3908580-supplementary.pdf]

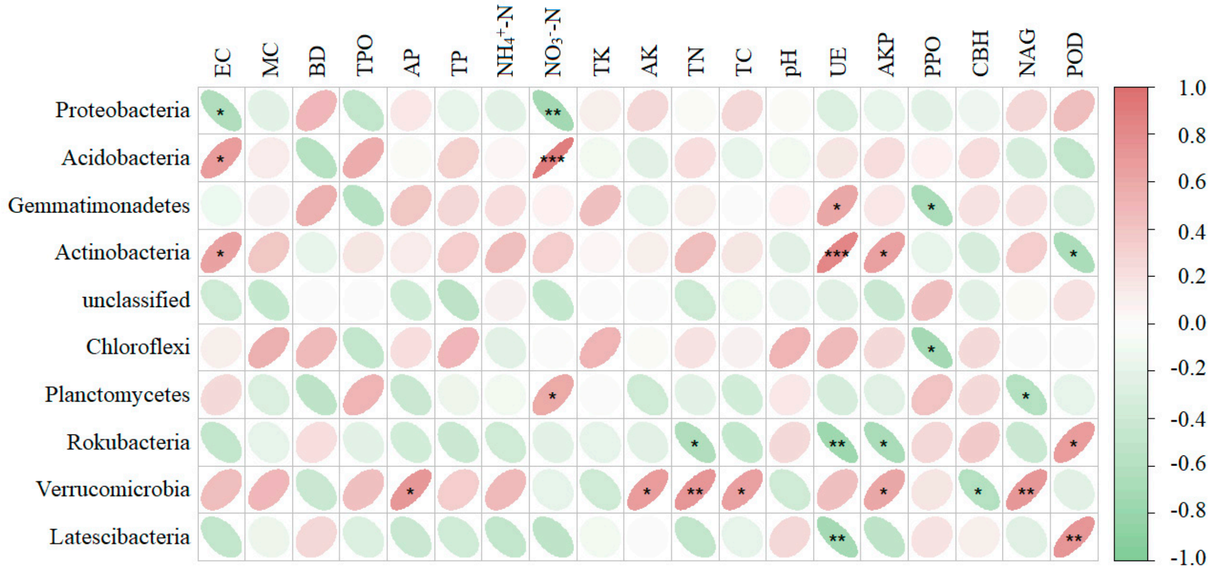

Figure S1 Correlation between dominant bacterial taxa (phylum level) and soil physicochemical properties and enzyme activities.

Not： *, $p < 0.05$; **, $p < 0.01$;***, $p < 0.001$. The same below.

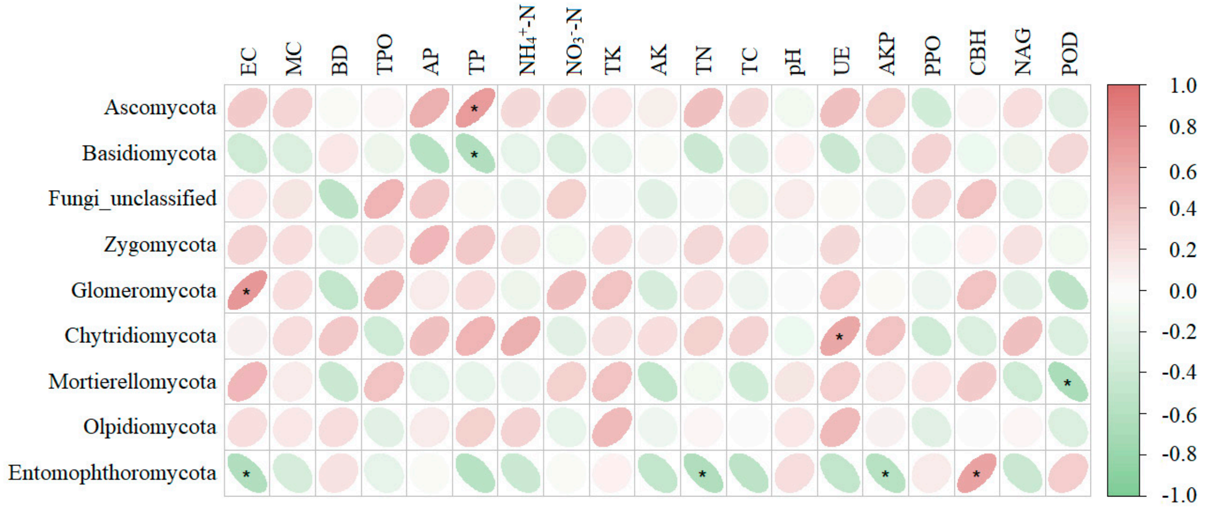

Figure S2 Correlation between dominant fungal taxa (phylum level) and soil physicochemical properties and enzyme activities.

Table S1 PERMANOVA test of soil microbial community species composition under different agroforestry models.

	Pairs	Df	SS	F	R^2	*p*
Bacteria	CPH vs CPL	1	0.27	1.16	0.22	0.12
	CPH vs CPS	1	0.20	1.07	0.21	0.20
	CPH vs CK	1	0.25	1.19	0.23	0.12
	CPL vs CPS	1	0.27	1.23	0.23	0.12
	CPL vs CK	1	0.34	1.38	0.26	0.12
	CPS vs CK	1	0.25	1.31	0.25	0.12
Fungus	CPH vs CPL	1	0.36	1.73	0.30	0.12
	CPH vs CPS	1	0.49	1.78	0.31	0.12
	CPH vs CK	1	0.31	1.10	0.22	0.20
	CPL vs CPS	1	0.71	3.55	0.47	0.12
	CPL vs CK	1	0.47	2.28	0.36	0.12
	CPS vs CK	1	0.63	2.318	0.37	0.12

Not：Df，degree of freedom; SS，Total Sum of Squares.

Table S2 Soil physicochemical characteristics under different agroforestry model.

Agroforestry model		CPH	CPS	CPL	CK
Bacteria	Shannon-Wiener diversity index	9.02±0.05a	9.29±0.32a	8.78±0.14a	8.99±0.16a
	Simpson diversity index	0.997±0.00a	0.998±0.00a	0.997±0.00a	0.999±0.00a
	Chaol diversity index	748.53±34.76a	1031.00±322.14a	627.52±60.71a	760.60±94.83a
Fungal	Shannon-Wiener diversity index	5.60±0.42a	6.16±0.39a	3.95±1.08a	4.52±0.50a
	Simpson diversity index	0.928±0.02ab	0.952±0.01a	0.760±0.10b	0.866±0.03ab
	Chaol diversity index	774.45±30.88a	671.24±32.63ab	542.99±67.27b	565.46±57.01b
	EC（ds·m^{-1}）	1.34±0.03ab	1.27±0.01bc	1.38±0.03a	1.22±0.02c
	MC（%）	18.48±0.52ab	19.34±0.38a	18.62±0.32ab	17.63±0.59b
	BD（g·cm^{-3}）	1.43±0.01c	1.54±0.01a	1.44±0.01c	1.50±0.01b
	TPO（%）	46.04±0.22a	42.01±0.25c	45.66±0.22a	43.51±0.54b
	AP（mg·kg^{-1}）	9.99±1.23a	10.42±0.85a	8.43±1.05a	6.76±1.86a
	TP（g·kg^{-1}）	1.72±0.08ab	1.98±0.21a	1.84±0.14a	1.29±0.15b
	NH_4^+-N（mg·kg^{-1}）	0.69±0.21a	0.61±0.30a	0.38±0.08a	0.14±0.06a
	NO_3^--N (mg·kg^{-1})	2.34±0.70b	1.76±0.45b	6.08±1.32a	2.53±0.49b
	TK (g·kg^{-1})	3.18±0.24b	4.15±0.19ab	4.47±0.46a	4.34±0.46ab
	AK（mg·kg^{-1}）	244.27±31.88a	223.70±35.33a	78.37±14.59b	52.93±4.56b
	TN (g·kg^{-1})	0.94±0.07a	0.94±0.02a	0.87±0.02a	0.58±0.06b
	TC (g·kg^{-1})	16.40±0.93a	16.54±0.24a	14.70±0.06ab	13.63±0.58b
	C: N	17.54±0.30a	17.67±0.21a	16.98±0.41a	23.80±1.51b
	pH	7.80±0.25a	8.17±0.03a	8.11±0.04a	8.20±0.03a
	UE (U·g^{-1})	893.89±8.73bc	1018.64±38.73a	974.79±31.41ab	831.75±19.14c
	AKP (U·g^{-1})	10.77±0.33a	11.72±0.30a	10.86±0.56a	8.48±0.28b
	PPO (U·g^{-1})	12.22±0.36a	9.76±0.25b	10.85±0.36ab	11.51±0.81a
	CBH (U·g^{-1})	4.46±0.26b	5.43±0.52b	11.18±1.63a	12.54±1.22a
	NAG (U·g^{-1})	6.59±0.79a	7.14±0.22a	3.13±0.45b	2.39±0.34b
	POD (U·g^{-1})	4.60±0.57ab	4.91±0.44ab	3.38±0.67b	5.35±0.29a
	BG (U·g^{-1})	129.58±17.00a	131.77±11.28a	24.84±3.75b	150.78±9.53a

Not：Mean ± Standard Error.

Table S3 Kruskal-Wallis test-based analysis of differential abundance in bacterial phyla.

Phylum	p value	q value	Significance
Acidobacteria	0.02	0.23	*
GN04	0.03	0.23	*
Verrucomicrobia	0.03	0.23	*
Omnitrophicaeota	0.04	0.23	*
Actinobacteria	0.04	0.23	*
WS3	0.05	0.23	*
Zixibacteria	0.06	0.23	ns
Bacteroidetes	0.06	0.23	ns
Planctomycetes	0.08	0.23	ns
Chloroflexi	0.08	0.23	ns
Chlamydiae	0.09	0.23	ns
Latescibacteria	0.09	0.23	ns
Proteobacteria	0.09	0.23	ns
Nitrospirae	0.09	0.23	ns
Gemmatimonadetes	0.11	0.23	ns
Dependentiae	0.11	0.23	ns
Rokubacteria	0.11	0.23	ns
Dadabacteria	0.15	0.30	ns
Elusimicrobia	0.17	0.30	ns
Hydrogenedentes	0.17	0.30	ns
GAL15	0.22	0.37	ns
unclassified	0.31	0.49	ns
Armatimonadetes	0.37	0.54	ns
Calditrichaeota	0.39	0.54	ns
Spirochaetes	0.39	0.54	ns
Entotheonellaeota	0.40	0.54	ns
Bacteriaunclassified	0.48	0.60	ns
BRC1	0.53	0.60	ns
OD1	0.53	0.60	ns
TM7	0.53	0.60	ns
Fibrobacteres	0.53	0.60	ns
Cyanobacteria	0.55	0.60	ns
Firmicutes	0.74	0.78	ns
Patescibacteria	0.88	0.90	ns
TM6	0.98	0.98	ns

Not： *, $p<0.05$, The same below.

Table S4 Kruskal-Wallis test-based analysis of differential abundance in fungal phyla.

Phylum	p value	q value	Significance
Glomeromycota	0.04	0.34	*
Entomophthoromycota	0.09	0.40	ns
Ascomycota	0.19	0.52	ns
Basidiomycota	0.28	0.52	ns
Chytridiomycota	0.32	0.52	ns
Fungi_unclassified	0.35	0.52	ns
Olpidiomycota	0.49	0.60	ns
Mortierellomycota	0.53	0.60	ns
Zygomycota	0.76	0.76	ns
